# Supplementary material for: Characterization of Epithelial–Mesenchymal and Neuroendocrine Differentiation States in Pancreatic and Small Cell Ovarian Tumor Cells and Their Modulation by TGF-β1 and BMP-7
Source: Cells. 2024 Dec 5;13(23):2010. doi: 10.3390/cells13232010 (PMC11640004; doi:10.3390/cells13232010)
Supplement: Supplementary file 1 [file cells-13-02010-s001.zip › cells-3130552-supplementary.pdf]

Supplementary Figure S1

STR fragment analysis of cell lines

	Bon		BxPC3		HPDE K-SFM/RPMI		MiaPaCa2		NT3		Panc1		SCCOHT-1		Bin-67	
STR fragment	allele 1	allele 2	allele 1	allele 2	allele 1	allele 2	allele 1	allele 2	allele 1	allele 2	allele 1	allele 2	allele 1	allele 2	allele 1	allele 2
Amelogenin	X-chrom.	Y-chrom.	X-chrom.		X-chrom.		X-chrom.		X-chrom.	Y-chrom.	X-chrom.		X-chrom.		X-chrom.	
D10S1248	15	16	13	17	14	17	14	15	14		14		13		13	
D12S391	19.1		19.3	20	21	22	19		18.3	22	22		20	21	18	20
D16S539	10	11	9	11	11	13	10	13	8	12	11		9	13	12	
D18S51	12		12		13	18	12		12	18	12		13		13	19
D19S433	12	15.2	13	16.2	12	14	15		14	16	11	16	15.2	16	14	15.2
D1S1656	13		16	17.3	16.3	17.3	15	17.3	17	18.3	12	14	12	16	15	17.3
D21S11	28	33.2	29		30	33	29	31.2	28	29	28		28	31.2	30	
D22S1045	19		17		16		16		16		16		15	16	16	
D2S1338	16	23	17	19	17	20	25		18	24	23	24	17		17	25
D2S441	10	14	12	14	10	11	14		11	14	10	14	11.3	14	8	10
D3S1358	17		14	16	16		16		18		17		15	17	15	19
D8S1179	10	12	13		14	16	16		15		14	15	13	14	14	
FGA	23		20	21	21	22	22		20	26	21		25	26	22	23
SE33	29.2		28.2		18	25.2	16		22.2	26.2	12	17	17	18	16	30.2
TH01	8		9		9.3		9	10	6	9.3	7	8	7	8	6	9.3
VWA	19		14	18	14	17	15		16	17	15		14	16	14	18

Figure S1. STR fragment analysis of the cell lines used in this study. Authentication of the indicated cell lines was performed by short tandem repeat (STR) fragment analysis using the GenomeLab human STR primer set purchased from Beckman Coulter Inc.

Supplementary Figure S2

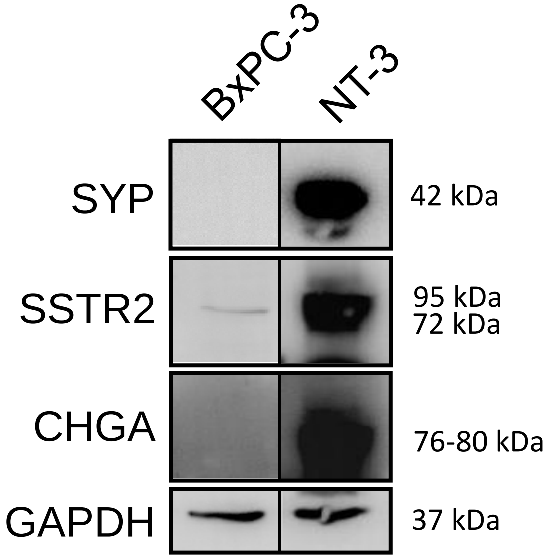

Figure S2. Immunoblot analysis of the indicated NED markers in BxPC-3 cells. Human insulinoma NT-3 cells were used as a positive control. Detection of GAPDH was used as control for equal loading.

Supplementary Figure S3

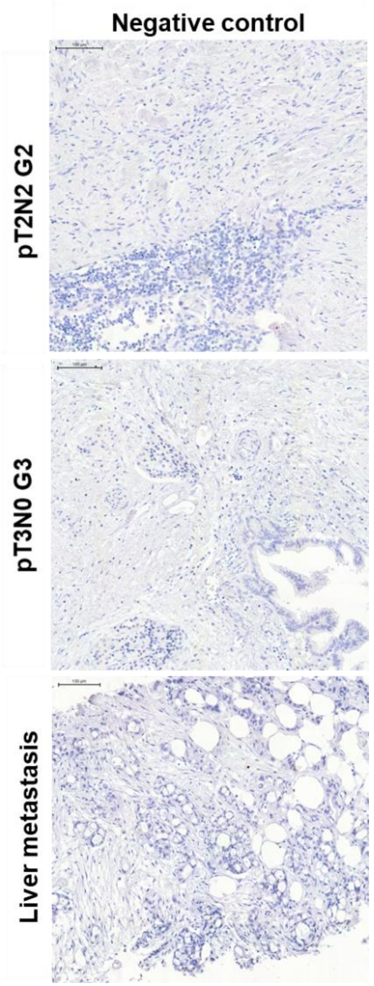

Figure S3. Negative controls for IHC. Tissue sections adjacent to those shown in Figure 3 were incubated with the respective IgG rather than the specific primary antibody (anti-CHGA, anti-SYP or anti-SSTR2) and subsequently processed along with these as described in the Material and Methods section.

Supplementary Figure S4

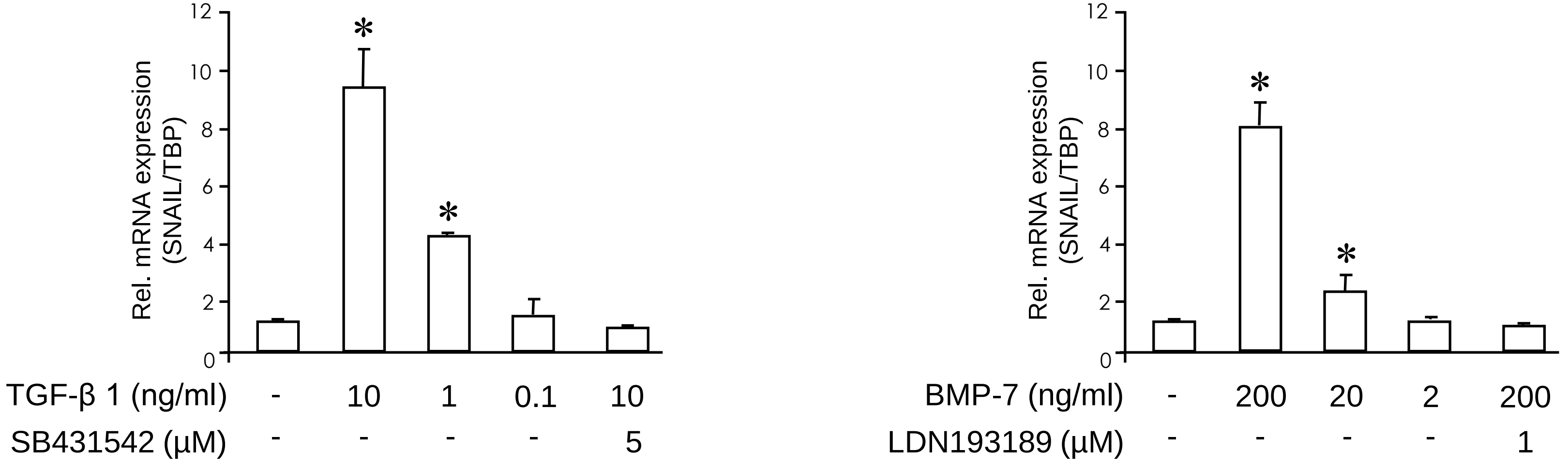

Figure S4. Dose-response curve of induction of *SNAI1* by TGF-β1 or BMP-7 and effect of SB431542 and LDN193189 in PANC-1 cells. PANC-1 cells were treated for 24 h with either vehicle (-) or the indicated concentrations of either TGF-β1 (left panel) or BMP-7 (right panel) in the absence or presence of the TGF-β/ALK5 inhibitor SB431542 (5 µM) or the BMP/ALK2 inhibitor LDN193189 (1 µM), respectively. Cells were then subjected to qPCR analysis of SNAIL, and the housekeeping gene TATA box binding protein (TBP) to account for small differences in cDNA input. Data are the normalised mean ± SD of triplicate wells. The asterisks (∗) indicate significant differences relative to vehicle-treated cells.

Supplementary Figure S5

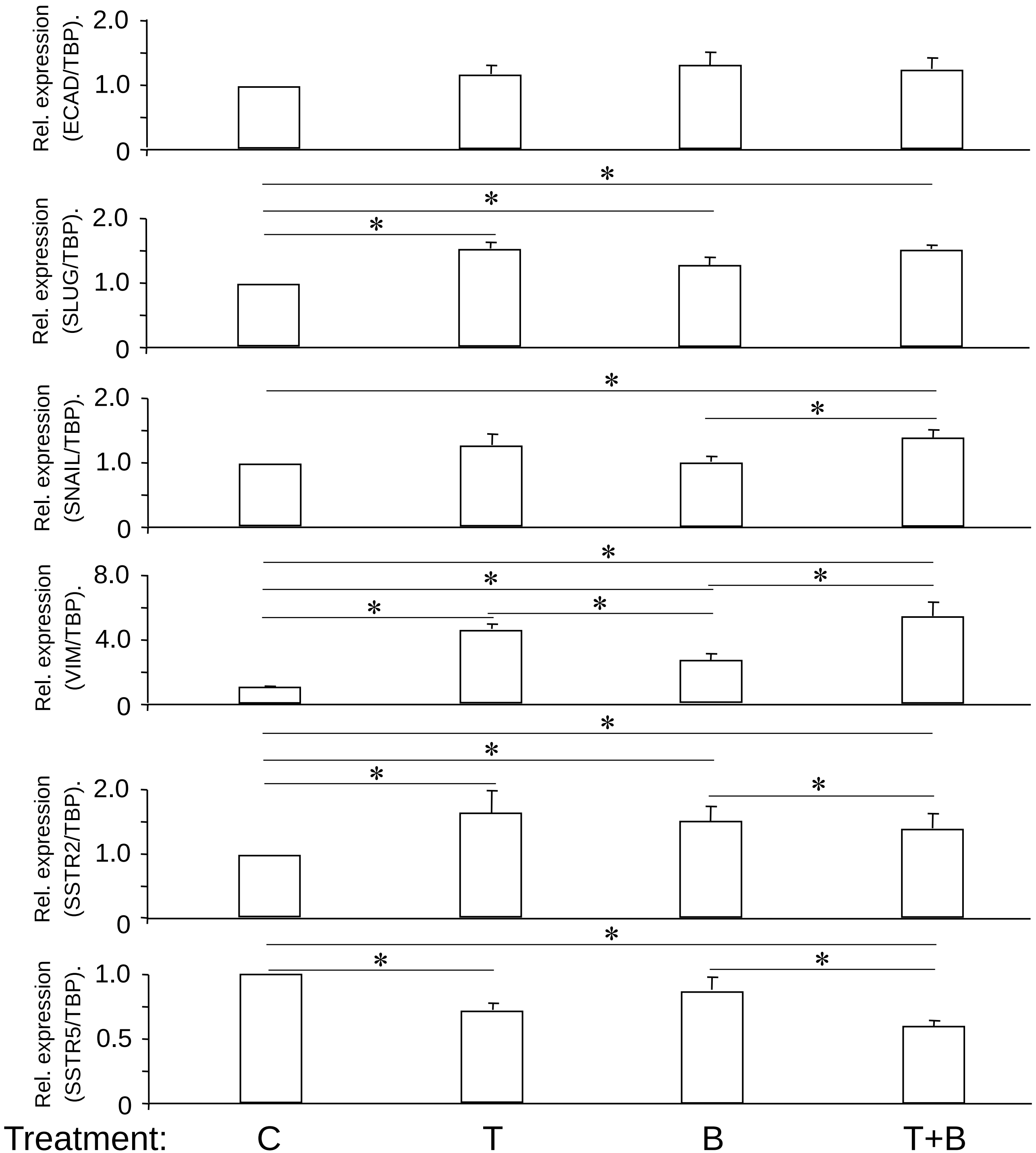

Figure S5. Effect of TGF-β1 and BMP-7 treatment on EMT and NED marker expression in BxPC-3 cells. BxPC-3 cells were seeded in 24-well plates on day 1 and were treated on day 2 for 24 h with either vehicle (Control, C) TGF-β1 (T, 10 ng/ml) or BMP-7 (B, 200 ng/ml), singly or in combination (T+B). Following lysis, cells were subjected to qPCR analysis of the indicated genes (see ordinata). TBP was also amplified to account for small differences in cDNA input. Data are the normalized mean ± SD of triplicate wells. The asterisks (*) indicate significant differences ($p<0.05$, two-tailed unpaired Student's *t*-test).

Supplementary Figure S6

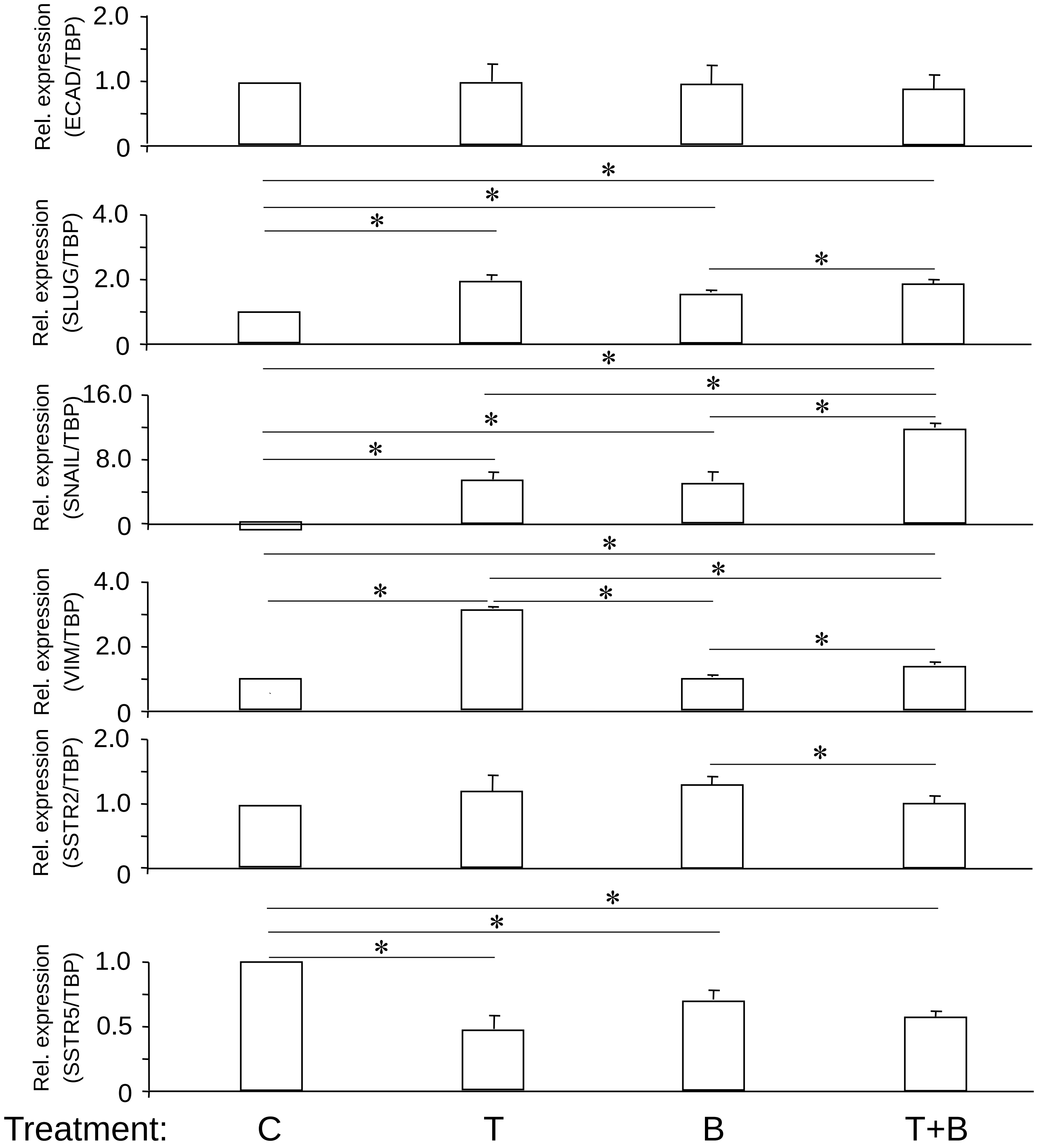

Figure S6. Effect of TGF-β1 and BMP-7 treatment on EMT and NED marker expression in HPDE cells. HPDE cells were seeded in 24-well plates on day 1 and were treated on day 2 for 24 h with either vehicle (Control, C), TGF-β1 (T, 10 ng/ml) or BMP-7 (B, 200 ng/ml), singly or in combination (T+B). Following lysis, cells were subjected to qPCR analysis of the indicated genes (see ordinata). TBP was also amplified to account for small differences in cDNA input. Data are the normalized mean ± SD of triplicate wells. The asterisks (*) indicate significant differences ($p<0.05$, two-tailed unpaired Student's *t*-test).

Supplementary Figure S7

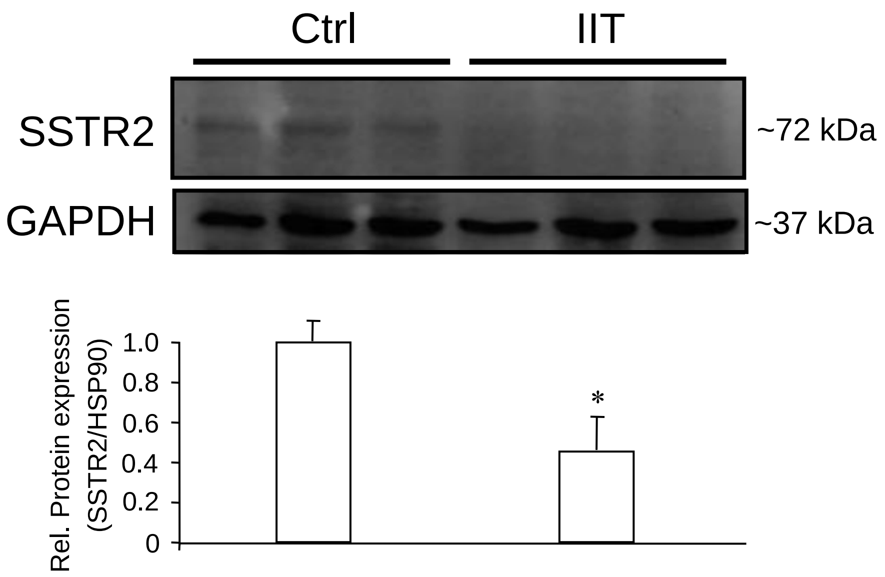

Figure S7. Immunoblot analysis of SSTR2 in PANC-1 cells induced to undergo MET. Parental cultures of PANC-1 cells were exposed to a cocktail of three cytokines, IL-1β, IFN-γ and TNF-α (IIT), or medium without these cytokines (Ctrl, three parallel wells each), for 72 h and subsequently subjected to immunoblot analysis of SSTR2, and GAPDH as a control for equal protein loading. The graphs below the blots denote the results of densitometric band quantification (SSTR2/GAPDH). The data shown represent the mean ± SD of three parallel samples. The asterisk (*) denotes a significant difference (p=0.017).
